# Nutrient solution strength affects growth, physiology, biochemistry and fruit quality of Korean strawberry ‘Kuemsil’ in a recycling hydroponic system

**DOI:** 10.3389/fpls.2025.1685755

**Published:** 2025-12-12

**Authors:** Mewuleddeg Zebro, Jeong-Hyeon Baek, Minkyung Kim, Youngae Jeong, M. G. Rabbani, Ki-Young Choi

**Affiliations:** 1 Agriculture and Life Sciences Research Institute, Kangwon National University, Chuncheon, Republic of Korea; 2 Department of Smart farm and agricultural industry, Kangwon National University, Chuncheon, Republic of Korea; 3 Department of Horticulture and Plant Science, Jimma University, Jimma, Ethiopia; 4 Department of agriculture and industries, Kangwon National University Graduate School, Chuncheon, Republic of Korea

**Keywords:** fruit quality, hydroponics, photosynthetic rate, peroxidase, catalase

## Abstract

**Introduction:**

Determining the optimal nutrient solution strength is critical for sustainable strawberry production in hydroponics. This study investigated the effects of nutrient solution strength on growth, physiological performance, antioxidant activity, and fruit quality of the strawberry cultivar ‘Kuemsil’ grown in a recycling hydroponic system.

**Methods:**

Four nutrient solution strengths were tested: one-third (1/3S), half (1/2S), two-thirds (2/3S), and full strength (1S) of the Japan Horticultural Research Institute solution, using coconut coir substrate in a greenhouse for 175 days from September 2024 to May 2025. Plant growth traits, photosynthetic parameters, pigment concentrations, antioxidant activity, and fruit quality attributes were measured.

**Results:**

Results showed that the 2/3S treatment significantly enhanced vegetative growth, photosynthetic rate, stomatal conductance, and pigment content. Furthermore, the 2/3S treatment improved antioxidant capacity, including total phenolic content, total flavonoid content, DPPH radical scavenging activity, and ferric reducing antioxidant power. Moreover, antioxidant enzyme activities, such as peroxidase and catalase, were substantially higher under the 2/3S treatment compared to the other treatments. Fruit weight, firmness, soluble solids content, and mineral contents (potassium, magnesium, phosphorus) were also highest under 2/3S, whereas 1/3S consistently produced the lowest values. In contrast, the 1/3S treatment resulted in higher values for absorbed energy per reaction center (ABS/RC) and trapped energy per reaction center (TRo/RC). These increases suggest that the reaction centers were subject to greater excitation pressure and retained more of the absorbed energy under reduced nutrient strength.

**Discussion:**

Based on these findings, the 2/3S treatment provided the best overall balance of growth, physiological performance, and fruit quality. Therefore, applying a two-thirds-strength nutrient solution is optimal for improving both productivity and fruit quality of the ‘Kuemsil’ strawberry cultivar under recycling hydroponic conditions.

## Introduction

Strawberry (*Fragaria × ananassa*) is one of the most economically important and nutritionally valuable berry crops worldwide. It is well-known for its pleasant flavor and high antioxidant content, making it popular for both fresh consumption and processed foods. Strawberry cultivation has become an essential component of horticultural industries globally. In 2023, global strawberry production reached approximately 10.48 million tons over 434,977 hectares (FAOSTAT, 2023). In South Korea, strawberries are a major horticultural crop, with the most commonly grown local varieties being ‘Seolhyang,’ ‘Maehyang,’ ‘Kuemsil’ and ‘Goha.’ The cultivation season for most varieties begins in September and continues through May of the following year. In 2023, South Korea produced approximately 177,682 tons of strawberries across 5,842 hectares (FAOSTAT, 2023), with about 99.5% grown under greenhouse conditions (MAFRA, 2023).

In South Korea, the use of hydroponic systems for cultivating strawberries is increasing due to a growing interest in sustainable farming. By 2020, the area allocated for hydroponically grown strawberries had reached 1,596 hectares (Choi et al., 2022). This figure increased to 3,009 hectares by 2022, accounting for 56.3% of the total hydroponic cultivation area (Kim et al., 2024). Hydroponic systems are divided into open and recycling types. Open systems, which are still common in South Korea, discharge nutrient solutions after each irrigation cycle. In contrast, recycling systems retain the nutrient solution, which helps lower costs, minimize nutrient waste and enhance sustainability (Kim et al., 2023). Moreover, recycling systems are considered more eco-friendly, as they prevent the release of excess chemicals into the environment. Despite these benefits, the effectiveness of recycling systems heavily relies on precise and efficient nutrient management. Strawberries cultivated in hydroponic systems require different nutrient strengths based on growth conditions, developmental stages and cultivar types. Lieten (1995) reported that strawberries commonly flourish within an electrical conductivity (EC) range of 1.0 to 1.4 dS·m^-1^. However, more recent studies have suggested higher optimal EC levels. For example, Kumar and Saini (2020) proposed an EC range of 1.8 –2.0 dS·m^-1^ during vegetative growth and 1.8 –2.5 dS·m^-1^ during fruit development. Furthermore, D’Anna et al. (2003) found that an EC of 2.5 dS·m^-1^ was optimal for achieving superior growth and fruit quality in the ‘Pudla’ cultivar. In contrast, Pourhosseini (2021) indicated that optimal vegetative growth occurred at 1.7 dS·m^-1^, while the highest fruit quality was recorded at 1.3 dS·m^-1^. These findings underscore the importance of optimizing nutrient solution strength for specific cultivars and growth stages. Moreover, in recycling systems, the continuous reuse of nutrient solutions can lead to imbalances that affect plant health and fruit development. Therefore, it is essential to regularly monitor and adjust the concentration and composition of the nutrient solution to maintain healthy growth, enhance yield, and ensure high fruit quality.

In hydroponic systems, the physiological and biochemical responses to nutrient levels are crucial indicators of plant health. Stomatal conductance, which regulates gas exchange and water use efficiency, is highly sensitive to nutrient availability. When nutrients are insufficient, stomatal conductance declines, restricting CO_2_ uptake and thereby diminishing photosynthetic efficiency and plant growth (Jiang et al., 2023). Essential macronutrients like nitrogen, phosphorus, potassium and magnesium play critical roles in photosynthesis, chlorophyll production, ATP synthesis and the Calvin cycle (Lu et al., 2025). Variations in these nutrients significantly affect plant physiological processes. For example, nitrogen deficiency often results in reduced chlorophyll content, leading to chlorosis and decreased photosynthetic capacity (Hamann et al., 2020). Moreover, nutrient stress can elevate respiration rates, indicating a metabolic shift from growth to stress response, which increases energy demands and oxidative stress (Shirko et al., 2018). Moreover, nutrient deficiencies may lead to an accumulation of reactive oxygen species (ROS), causing cellular damage. In response, plants activate an antioxidant defense system that includes both enzymatic and non-enzymatic components. Enzymatic antioxidants such as superoxide dismutase (SOD), peroxidase (POD) and catalase (CAT) mitigate ROS by converting them into less harmful substances. These enzymes have been found to increase significantly in strawberry plants under nutrient-deficient conditions (Jiang et al., 2023). Non-enzymatic antioxidants, including phenolic compounds and flavonoids also contribute to ROS scavenging while enhancing fruit quality attributes like flavor, color and nutritional value (Perin et al., 2019). The production of these compounds often rises in response to nutrient stress, underscoring their role in mitigating stress and improving fruit quality (Wang et al., 2023).

Given these complicated responses, several studies have examined nutrient solution management in hydroponic strawberry cultivation. Jun et al. (2013) evaluated EC levels ranging from 0.6 to 1.8 dS·m^−^¹ and found that moderate EC levels between 0.8 and 1.2 dS·m^−^¹ significantly improved the growth, yield, and fruit traits of the ‘Maehyang’ variety. Similarly, Lee et al. (2015) tested EC levels from 0.5 to 2.0 dS·m^−^¹ and discovered that a moderate EC of 1.0 dS·m^−^¹ enhanced growth in ‘Goha’, while higher levels of 2.0 dS·m^−^¹ negatively impacted the yield of ‘Albion’. In a related study, Lee J. H. et al. (2017) reported that both the composition of the nutrient solution and the resulting EC of 1.0 dS·m^−^¹ influenced the growth, photosynthetic capacity, and productivity of ‘Maehyang’ in coir-based hydroponics. Schiavon et al. (2022) also showed that nutrient solution formulations consistent with moderate EC conditions between 1.2 and 1.5 dS·m^−^¹ significantly affected stolon and propagule development in ‘Aromas’ and ‘Camarosa’, although the effects on seedling quality were minimal. Additionally, Sarooshi and Cresswell (1994) assessed EC levels from 2 to 4 dS·m^−^¹ in ‘Torrey’ and found that a level of 2 dS·m^−^¹ improved both yield and quality. While these studies highlight the importance of nutrient solution management in strawberries, most have focused on open hydroponic systems, where nutrient and water inputs are not recycled. To the best of our knowledge, no comprehensive study has simultaneously evaluated the growth, physiology, biochemical attributes, antioxidant properties, and fruit quality of strawberries under different nutrient solution strengths in a recycling hydroponic system. Moreover, responses to nutrient strength vary across cultivars, emphasizing the need for cultivar-specific evaluations. As a result, this study focused on ‘Kuemsil’, an interspecific hybrid derived from ‘Maehyang’ × ‘Seolhyang’. ‘Kuemsil’ is notable for its strong disease resistance, early harvest potential, firm fruit texture, and high consumer acceptance (Yoon et al., 2020). However, its physiological and fruit quality responses to varying nutrient solution strengths under recycling hydroponic conditions have not been systematically investigated. Therefore, this study aimed to determine how different nutrient solution strengths influence the growth, physiological performance, biochemical properties and fruit quality of ‘Kuemsil’ strawberry in a recycling hydroponic system.

## Materials and methods

### Plant material and treatment condition

The experiment was conducted in a Venlo-type greenhouse at Kangwon National University, located in Chuncheon, Gangwon Province, South Korea (37.8690° N, 127.7450° E). Seedlings of the strawberry variety ‘Kuemsil’ (*Fragaria × ananassa*) were obtained from a commercial strawberry farm in PyeongChang, South Korea. The seedlings had an average of 4 leaves, 18.5 cm petiole length, 9.8 cm leaf length, 7.2 cm leaf width, and 10.5 mm crown diameter. On September 20, 2024, seedlings were transplanted into 100% dust coconut coir substrate (Duck Yang Coco, Duck Yang Green, Sri Lanka) with 15 cm × 20 cm × 100 cm (H × W × L). The substrate had been pre-washed and nutrient-buffered to eliminate residual salts and to stabilize pH levels. Twelve plants were transplanted per slab, spaced 20 cm apart to maintain uniform density. Each slab had six strawberry plants on the right side and six on the left side. Following transplantation, the plants underwent a 25-day acclimatization period. Environmental parameters, including temperature, relative humidity, and integrated solar radiation, were automatically regulated using the Ridder CX500 climate control system (Ridder HortiMax, Harderwijk, The Netherlands). During the growing period, the daytime and nighttime temperatures averaged 19.9°C and 13.0°C, respectively, with relative humidity values of 74.8% during the day and 83.4% at night. Integrated solar radiation ranged from 801.5 to 2078.8 J·cm^−^², with an average of 1292.9 J·cm^−^². After 25 days of transplanting, experimental treatments began on November 15, 2024, and continued until May 9, 2025.

The experiment assessed four strengths of a standardized nutrient solution of the Japan Horticultural Research Institute: one-third strength (1/3S), half strength (1/2S), two-thirds strength (2/3S), and full strength (1S). The experiment was arranged in a randomized complete block design (RCBD) and each replicate includes one slab with 12 plants, and three replicates were used per treatment. The specific content of the full-strength solution is detailed in **Table 1**. Micronutrient concentrations were supplied at the 1S level across all treatments. During the cultivation period, a recycling hydroponic system was employed to deliver nutrient solutions efficiently and sustainably. The nutrient solution was refilled to its original strength every 10 days, and sometimes it was completely replaced when needed. Electrical conductivity (EC), pH, and concentrations of key ions, including total nitrogen, phosphorus, potassium, and calcium, are shown in **Table 2**. Irrigation was scheduled daily from 09:00 a.m. to 14:20, with the nutrient solution applied in nine evenly spaced cycles at 40-minute intervals. Under normal weather conditions, each plant received a total of 450 mL of solution per day. On days with low solar radiation or cloud cover, the irrigation volume was adjusted to 250 mL per plant to account for reduced evapotranspiration.

**Table 1:** Contents of the full-strength of Japan Horticultural Research Institute nutrient solution used in the experiment.

Macronutrient	mg·L^-1^	Micronutrient	mg·L^-1^
5[Ca(NO_3_)_2_·2H_2_O]NH_4_NO_3_	864	Fe-EDTA	23.07
KNO_3_	676.7	H_3_BO_3_	2.86
MgSO_4_·7H_2_O	492	MnSO_4_·H_2_O	1.54
KH_2_PO_4_	176.8	ZnSO_4_·7H_2_O	0.22
NH_4_NO_3_	40	CuSO_4_·5H_2_O	0.08
		Na_2_MoO_4_·2H_2_O	0.03

**Table 2:** The pH, EC, total nitrogen, phosphorus, potassium and calcium contents of each treatment.

Strengths	pH	EC (dS·m^-1^)	Total nitrogen	Phosphorus	Potassium	Calcium
			mg·L^-1^			
1/3S	6.2	1.1	101.6	15.1	139.7	78.3
1/2S	6.0	1.5	142.2	21.0	208.7	121.3
2/3S	5.9	1.9	176.9	27.6	263.2	148.7
1S	5.8	2.5	270.9	42.0	346.8	190.5

### Determination of fruit and growth characteristics

Fruit-related parameters were collected from January 1 to March 30, 2025 (48 to 136 Days after treatment (DAT)). Fruit length and fruit width were assessed using a digital caliper, and fruit weight was recorded with an electronic balance (HS5200S, Hansang Instrument Co. Ltd., Korea). Soluble solids content (SSC) and titratable acidity (TA) were determined using a digital refractometer (PAL-1, Atago, Tokyo, Japan) and a digital acidity meter (PAL-Easy Acidity, Atago, Tokyo, Japan), respectively. Fruit firmness was measured using a fruit firmness tester (FR-5105, Lutron Electronic Enterprise Co., Ltd., Taiwan). Plant height was measured from the base of the plant to the tip of the longest leaf. Petiole length, leaf length, and leaf width were measured on the fully expanded third leaf using a ruler, with one leaf sampled from each of six plants. The number of leaves was counted manually, and crown diameter and petiole width were measured using a digital caliper (CD-20CPX, Mitutoyo Corp., Japan). These growth-related parameters were collected on May 9, 2025 (175 DAT).

### Physiological related measurements

All physiological parameters were measured on April 24, 2025 (160 DAT), except for chlorophyll fluorescence, which was measured on March 15, 2025 (120 DAT). Leaf gas exchange parameters such as net photosynthetic rate, transpiration rate, stomatal conductance, and intercellular CO2 concentration were measured using a portable photosynthesis system (LI-6800; LI-COR Inc., Nebraska, USA) on the third fully expanded leaf from the top of each plant. Measurements were conducted under controlled chamber conditions: temperature of 24°C, CO2 concentration of 400 μmol·mol^−^¹ and a photosynthetic photon flux density (PPFD) of 800 μmol·m^−^²·s^−^¹ within the 6 m² measurement area. Chlorophyll fluorescence was assessed on the same third leaf using a portable fluorometer (PAR-FluorPen FP 110/D; Photon Systems Instruments, Drásov, Czech Republic).

For the measurement of leaf relative water content (LRWC) and electrolyte leakage (EL), samples were collected from fully expanded third leaves. To determine LRWC, freshly detached strawberry leaves were immediately weighed to record their fresh weight (FW). The leaves were then submerged in distilled water inside sealed centrifuge tubes and kept at room temperature for 24 hours to ensure full hydration. After this period, the leaves were removed, gently blotted to remove surface moisture, and weighed again to obtain the turgid weight (TW). For dry weight (DW), the same leaf samples were oven-dried at 70°C up to we got a constant weight. Leaf relative water content was calculated using the formula described by Zygielbaum et al. (2009).


LRWC(%)=(FW−DW)/(TW−DW)×100

To assess membrane stability, 1 g of leaf tissue was cut into 0.5 cm segments and placed in 25 mL of deionized water. The samples were incubated on a rotary shaker for 12 hours at room temperature, after which the initial electrical conductivity (EL_i_) of the solution was measured using a conductivity meter (DS-71G, HORIBA Ltd., Japan). The samples were then autoclaved at 120°C for 15 minutes to completely release cellular electrolytes, followed by another 12-hour incubation on the shaker. The final conductivity (EL_f_) was then recorded. Electrolyte Leakage (EL) was calculated. Electrolyte leakage (EL) was determined by dividing the EL_i_ by the EL_f_.

To analyze chlorophyll content, 0.5 g per replication of leaf fresh weight was homogenized in 10 mL of 80% acetone. The homogenate was mixed thoroughly using a vortex mixer and sonicated for 20 minutes. Subsequently, the homogenates were centrifuged at 5,000 rpm for 20 minutes at 4°C using a LZ-1248R refrigerated centrifuge (Gyozen Co., Ltd, Gimpo, Korea). The resulting supernatant was carefully collected into new tubes and stored at –20°C in the dark until further analysis. Chlorophyll a, chlorophyll b, and total carotenoid contents were determined spectrophotometrically by measuring absorbance at 663, 645, and 470 nm, respectively. Concentrations were calculated from the absorbance values using the equations established by Wellburn and Lichtenthaler (1984) as the following:


Chl a (mg/g)=[12.7A663–2.69A645]×V×D/(1000×W)

Chl b (mg/g)=[22.9A645–4.68A663]×V×D/(1000×W)

Total Chl (mg/g)=[20.2A645+8.02A663]×V×D/(1000×W)

Carotenoids (mg/g)=[(1000A470–3.27(Chla–Chlb)]×V×D/(229×1000×W)

Where: V = volume of acetone used, D = dilution factor, W = weight of leaves used.

### Preparation of extracts and antioxidant activity assays

For antioxidant analysis, dried strawberry leaves and fruits were ground into a fine powder using a mortar and pestle. Leaf samples were collected on April 24, 2025 (160 DAT), while fruit samples were collected between January 1 and March 30, 2025 (48–136 DAT). A 0.1 g of the powdered sample was mixed with 7 mL of absolute methanol in a 50 mL Falcon tube for each replication. The mixture was vortexed to ensure complete mixing and then sonicated for 30 minutes. Following sonication, the sample was centrifuged at 4,500 rpm for 20 minutes at 4°C. The supernatant was collected, and the pellet was re-extracted with 80% methanol. Combined extracts were stored at –20°C for subsequent assays.

Total phenolic content (TPC) was quantified using a modified method of Zebro and Heo (2024a). In brief, 0.1 mL of the leaves and 0.2 fruit extract were mixed with 2.9 and 2.8 mL of Folin–Ciocalteu reagent, respectively, 0.75 mL of 20% sodium bicarbonate, and 1.25 mL of distilled water. The mixture was incubated in the dark at room temperature for 30 minutes, and absorbance was measured at 765 nm using a UV spectrophotometer (Shimadzu, UV-1800, Kyoto, Japan). Results were expressed as milligrams of gallic acid equivalent per gram of dry weight (mg GAE/g DW), based on a gallic acid calibration curve.

Total flavonoid content (TFC) was determined using a slightly modified protocol described by Zebro and Heo (2024a). A 0.1- and 0.2-mL aliquot of leaves and fruits extracts was mixed with 1.5 mL methanol, 0.1 mL of 1 M sodium acetate, 0.1 mL of 10% aluminum chloride, and 2.9 mL of distilled water. The reaction mixture was incubated for 30 minutes at room temperature in the dark. Absorbance was measured at 415 nm, and flavonoid content was expressed as milligrams of quercetin equivalent per gram of dry weight (mg QE/g DW), using a quercetin standard curve.

2,2-diphenyl-1-picrylhydrazyl (DPPH) radical scavenging capacity of the extracts was evaluated according to Lee et al. (2023). A 0.1 mL aliquot of leaves and fruits extracts was added to 2.9 and 2.8 mL of a 0.1 mM DPPH solution in methanol. The mixture was incubated at room temperature in the dark for 30 minutes. Absorbance was measured at 517 nm, and the scavenging activity (%) was calculated using the formula:


$$\text{DPPH scavenging activity }\left(\%\right)=\frac{\text{absorbance control }–\text{ absorbance sample}}{\text{absorbance control }}\times \;100$$

Ferric reducing antioxidant power (FRAP) activity was measured following the method of Zebro and Heo (2024a) with slight modifications. The FRAP reagent was freshly prepared by mixing 10 mM TPTZ (dissolved in 40 mM HCl), 300 mM acetate buffer and 20 mM FeCl_3_ in a 2.5:2.5:5 ratio. A 0.02 mL aliquot of leaves and 0.05 mL fruit extract were added to 2.98 and 2.95 mL of the FRAP, respectively, and incubated at 37°C for 4 minutes. Absorbance was then recorded at 593 nm. FRAP values were calculated based on a standard curve of Trolox (0–100 µg) and expressed as millimolar Trolox equivalents per gram of dry weight (mM TE/g DW).

### Antioxidant enzyme activities

Young strawberry leaves (0.5 g) at the same developmental stage and position were collected from each treatment group with three replications on April 24, 2025 (160 DAT). Samples were immediately frozen in liquid nitrogen and stored at –80°C until analysis. Enzymes were extracted using 100 mM phosphate buffer (pH 7.8). The activities of superoxide dismutase (SOD), peroxidase (POD), and catalase (CAT) were determined following the method described by Zhao et al. (2025).

### Analysis of fruit mineral contents

The mineral content of the fruits was analyzed to evaluate the effects of nutrient solution strength on fruit quality, using samples collected in March 2025 (136 DAT). Initially, the samples were freeze-dried until a constant weight was achieved. The dried samples were then ground using a mortar and pestle, and 0.5 g of the resulting powder was mixed with 10 mL of 50% perchloric acid and 1 mL of sulfuric acid. The mixture was thoroughly mixed and left overnight to allow for complete reaction. The following day, the samples underwent sequential heating until the solution became colorless. The heating steps are summarized in **Table 3**. After digestion, the samples were diluted 200-fold with ultrapure water, filtered using Whatman No. 6 filter paper (pore size 3 μm), followed by syringe filtration (pore size 0.45 μm). An additional 25-fold dilution was performed to match the concentration range of the standards. Mineral analysis was carried out using inductively coupled plasma optical emission spectrometry (Integra Dual, GBC, Australia). The instrument was calibrated with certified standards, and no further chemical treatment was applied prior to analysis. Magnesium, sodium, and potassium standards were purchased from Kanto Chemical Co., Inc. (Nihonbashi Honcho 3-Chome, Chuo-ku, Tokyo, Japan). Calcium and phosphate standards were obtained from Samchun Chemical (Pyeongtaek-si, Gyeonggi-do, Korea) and Supelco (Sigma-Aldrich, Darmstadt, Germany), respectively.

**Table 3:** Sequential heating conditions used for sample digestion in mineral analysis.

Step	Temperature (°C)	Duration (min)
1	88	120
2	180	30
3	350	120

### Statistical analysis

The experiment was conducted with RCBD to minimize environmental variability across the greenhouse. Growth characteristics were assessed with six biological replicates per treatment, fruit quality parameters with nine biological replicates, and chlorophyll content and leaf antioxidant capacity with six replicates. Physiological parameters (OJIP fluorescence, gas exchange, leaf relative water content, and electrolyte leakage), antioxidant enzyme activities, fruit antioxidant activity, and mineral content were analyzed using three replicates. Data were first examined for normality (Shapiro–Wilk test) and homogeneity of variances (Levene’s test) prior to statistical analysis. When assumptions were met, treatment means were compared using one-way analysis of variance (ANOVA), followed by Duncan’s multiple range test (DMRT) for *post-hoc* comparisons at a significance level of *p* < 0.05. When assumptions were not met, data were square-root transformed prior to analysis; if transformation did not normalize the data, non-parametric Kruskal–Wallis tests were applied. Data transformation in this study was performed for PCA standardization, as variables were measured on different scales. Standardization ensured that variables with larger numeric ranges did not dominate the principal components. Correlation analyses among growth, physiological, and biochemical parameters were conducted using Pearson’s correlation coefficients, with significance determined at *p* < 0.05 and *p* < 0.01. To further explore treatment-dependent clustering and to identify key traits contributing to variation among nutrient solution strengths, principal component analysis (PCA) was performed. Both correlation analysis and principal component analysis (PCA) were conducted using MetaboAnalyst 6 software (https://www.metaboanalyst.ca/). In PCA, eigenvalues greater than 1 were considered significant, and loadings plots were examined to determine the contribution of each variable to the principal components and their relationship with the treatments. All univariate analyses were performed in SPSS (version 26; IBM Corp., Armonk, NY, USA). The graphs were generated using Microsoft excel and MetaboAnalyst R. Data are presented as mean ± standard deviation of the mean, with exact *n* values provided in the respective figure and table legends.

## Results

### Growth and fruit characteristics

**Figure 1:**
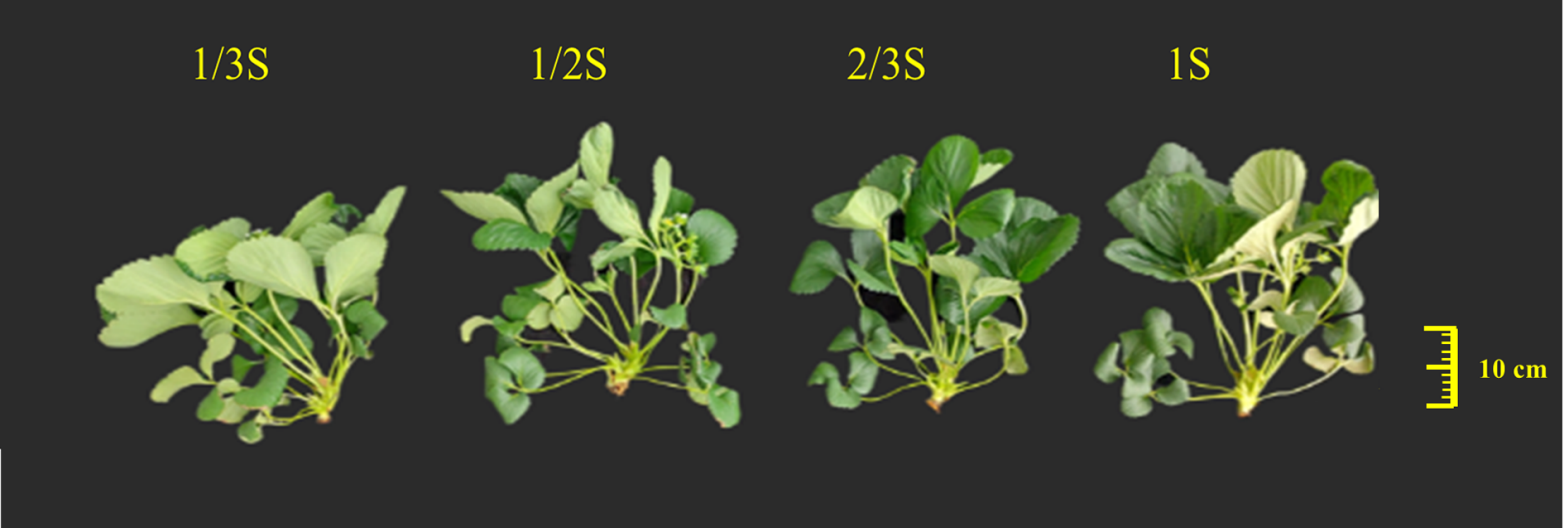
Growth performance of strawberry plants in response to varying nutrient solution strengths captured on May 9, 2025.

**Table 4** shows the effects of different nutrient solution strengths on the growth characteristics of strawberry plants. Treatments with 1/2S and 2/3S significantly enhanced plant growth compared to 1/3S and 1S. The number of leaves was significantly higher in the 2/3S treatment, while the 1/3S treatment showed the lowest leaf count (**Table 4**). Similarly, plant height was significantly greater in the 1/2S, 2/3S, and 1S treatments compared to 1/3S (**Table 4**, **Figure 1**). No significant differences were observed in petiole length across treatments, although petioles were numerically longer in the 2/3S treatment. Petiole diameter was significantly higher in the 1/2S and 2/3S treatments than in 1/3S and 1S. Similarly, leaf length was greater in the 1/2S, 2/3S, and 1S treatments, while the 1/3S treatment consistently showed lower values. For leaf width, higher values were observed in the 1/3S, 1/2S, and 2/3S treatments. Crown diameter was also significantly higher in the 1/2S, 2/3S, and 1S treatments compared to 1/3S. The evaluated treatments showed a significant difference in total fresh weight. Total fresh weight ranged from 134.1 to 150.4 g, with the lowest value recorded for the 1/3S treatment and the highest for the 2/3S treatment. Total dry weight ranged from 21.0 to 22.5 g, with the lowest value observed in the 1/3S treatment and the highest in the 2/3S treatment; however, no significant differences were noted among the treatments.

**Table 4:** Effects of different nutrient solution strengths on growth-related parameters of strawberry in recycling hydroponics at 175 DAT.

Treatments	Number of leaves	Plant height (cm)	Petiole length (cm)	Petiole diameter (mm)	Leaf length (cm)	Leaf width (cm)	Crown diameter (mm)	Total fresh weight (g/plant)	Total dry weight (g/plant)
1/3S	20.0^b^	38.4^b^	22.7^a^	4.3^b^	12.8^b^	11.7^a^	15.5^b^	134.1^b^	21.0^a^
1/2S	20.8^b^	39.5^ab^	22.5^a^	4.9^a^	14.0^a^	11.0^ab^	19.2^a^	140.5^ab^	21.3^a^
2/3S	23.3^a^	40.8^a^	23.9^a^	5.0^a^	14.1^a^	11.8^a^	18.9^a^	150.4^a^	22.5^a^
1S	21.3^b^	39.5^ab^	22.2^a^	4.6^b^	13.2^ab^	10.3^b^	18.8^a^	139.1^ab^	21.1^a^

Different letters within a column indicate statistically significant differences based on Duncan’s multiple range test (*p* < 0.05, n = 6).

Furthermore, fruit-related parameters were evaluated to assess the effects of varying nutrient solution strengths. The values for these parameters are shown in **Table 5**. While there was no significant difference in the number of fruits per plant across treatments, numerically, the 1S treatment recorded the lowest fruit count, while the 2/3S treatment had the highest. The average fruit weight per plant was highest under the 2/3S treatment, significantly exceeding that of the 1S treatment. However, the 1/3S and 1/2S treatments did not show significant differences from the 2/3S treatment. Total fruit weight per plant did not differ significantly among treatments; however, a numerical trend was observed in which the lowest values occurred under the 1S treatment and the highest under the 2/3S treatment. Total fruit weight in the 2/3S treatment was 10.5%, 3.5%, and 12.3% higher than in the 1/3S, 1/2S, and 1S treatments, respectively. Moreover, both fruit length and width were significantly higher in the 2/3S treatment compared to the other treatments. Fruit firmness was also significantly higher in the 2/3S treatment than in all other treatments. The soluble solid content (SSC), an important indicator of sweetness, was highest in the 2/3S and 1/2S treatments, while the 1/3S and 1S treatments showed significantly lower values. In contrast, titratable acidity (TA) was significantly higher in the 1S treatment compared to the others.

**Table 5:** Effects of different nutrient solution strengths on fruit related parameters of strawberry in recycling hydroponics collected from 48 to 136 DAT.

Treatments	Fruit number (no/plant)	Average fruit weight (g/fruit)	Total fruit weight (g/plant)	Fruit length (mm)	Fruit width (mm)	Fruit firmness (N/Ø3)	Soluble solids content (°Bx)	Titratable acidity (%)
1/3s	14.6^a^	17.1^ab^	248.9^a^	44.48^b^	35.03^b^	2.24^b^	11.51^c^	0.56^b^
1/2s	14.7^a^	18.3^ab^	268.4^a^	45.37^b^	35.21^b^	2.27^b^	12.55^a^	0.58^b^
2/3s	14.4^a^	19.3^a^	278.0^a^	48.07^a^	37.12^a^	2.53^a^	12.55^a^	0.59^b^
1S	13.7^a^	17.7^b^	243.9^a^	43.79^b^	34.23^b^	2.33^b^	12.07^b^	0.64^a^

Different letters within a column indicate statistically significant differences in means according to Duncan’s multiple range test (*p* < 0.05, n = 9).

### Chlorophyll fluorescence, leaf gas exchange and chlorophyll content

Different nutrient solution strengths resulted in significant differences in chlorophyll fluorescence (OJIP) and leaf gas exchange parameters. The maximum fluorescence ratio (Fm/Fo) and the variable fluorescence ratio (Fv/Fo) were significantly lower in the 1/3S treatment compared to the other treatments (**Table 6**). Similarly, the maximum quantum yield of PSII (Fv/Fm) was also significantly reduced in the 1/3S treatment (**Table 6**). The performance index on an absorption basis (PiAbs) was highest in the 1S treatment, followed by the 1/2S and 2/3S treatments, while the lowest value was observed in the 1/3S treatment (**Table 6**). The absorbed energy per reaction center (ABS/RC), trapped energy per reaction center (TRo/RC), electron transport rate per reaction center (ET_0_/RC), and energy dissipation per reaction center (DIo/RC) are key chlorophyll fluorescence parameters that describe how individual PSII reaction centers handle light energy. ABS/RC indicates the light energy absorbed by each reaction center. TR_0_/RC shows the portion of absorbed energy trapped for photochemistry. ET_0_/RC reflects the efficiency of energy transfer through the electron transport chain. DI_0_/RC indicates energy dissipated as heat or fluorescence, protecting against excess excitation. Together, these parameters reveal the balance between light absorption, utilization, and protection in photosynthetic tissues. In this study, ABS/RC, TRo/RC, and ET_0_/RC were all significantly higher in the 1/3S treatment compared to the others (**Table 6**). DIo/RC was also significantly higher in the 1/3S treatment, whereas the 1S treatment had the lowest dissipation rate, although this was not significantly different from the rates in the 1/2S and 2/3S treatments (**Table 6**).

**Table 6:** Effects of different nutrient solution strengths on chlorophyll fluorescence (OJIP) in recycling hydroponics at 120 DAT.

Treatments	Fm/Fo	Fv/Fo	Fv/Fm	PiAbS	ABS/RC	TRo/RC	ET_0_/RC	DIo/RC
1/3S	6.06^b^	5.06^b^	0.83^b^	9.59^b^	1.56^a^	1.30^a^	0.95^a^	0.25^a^
1/2S	6.58^a^	5.58^a^	0.85^a^	13.20^ab^	1.32^ab^	1.12^ab^	0.84^ab^	0.20^b^
2/3S	6.74^a^	5.74^a^	0.85^a^	12.35^ab^	1.29^b^	1.10^ab^	0.79^b^	0.19^b^
1S	6.84^a^	5.84^a^	0.85^a^	16.49^a^	1.22^b^	1.04^b^	0.79^b^	0.17^b^

Different letters within a column indicate statistically significant differences according to Duncan’s multiple range test (*p* < 0.05, n = 3). where: Fm/Fo, ratio of maximum to minimum fluorescence; Fv/Fo, ratio of variable to minimum fluorescence; Fv/Fm, ratio of variable to maximum fluorescence; PiAbs, performance index on an absorption basis; ABS/RC, absorbed energy per reaction center; TRo/RC, trapped energy per reaction center; ET_0_/RC, electron transport per reaction center; DIo/RC, energy dissipation per reaction center.

**Figure 2:**
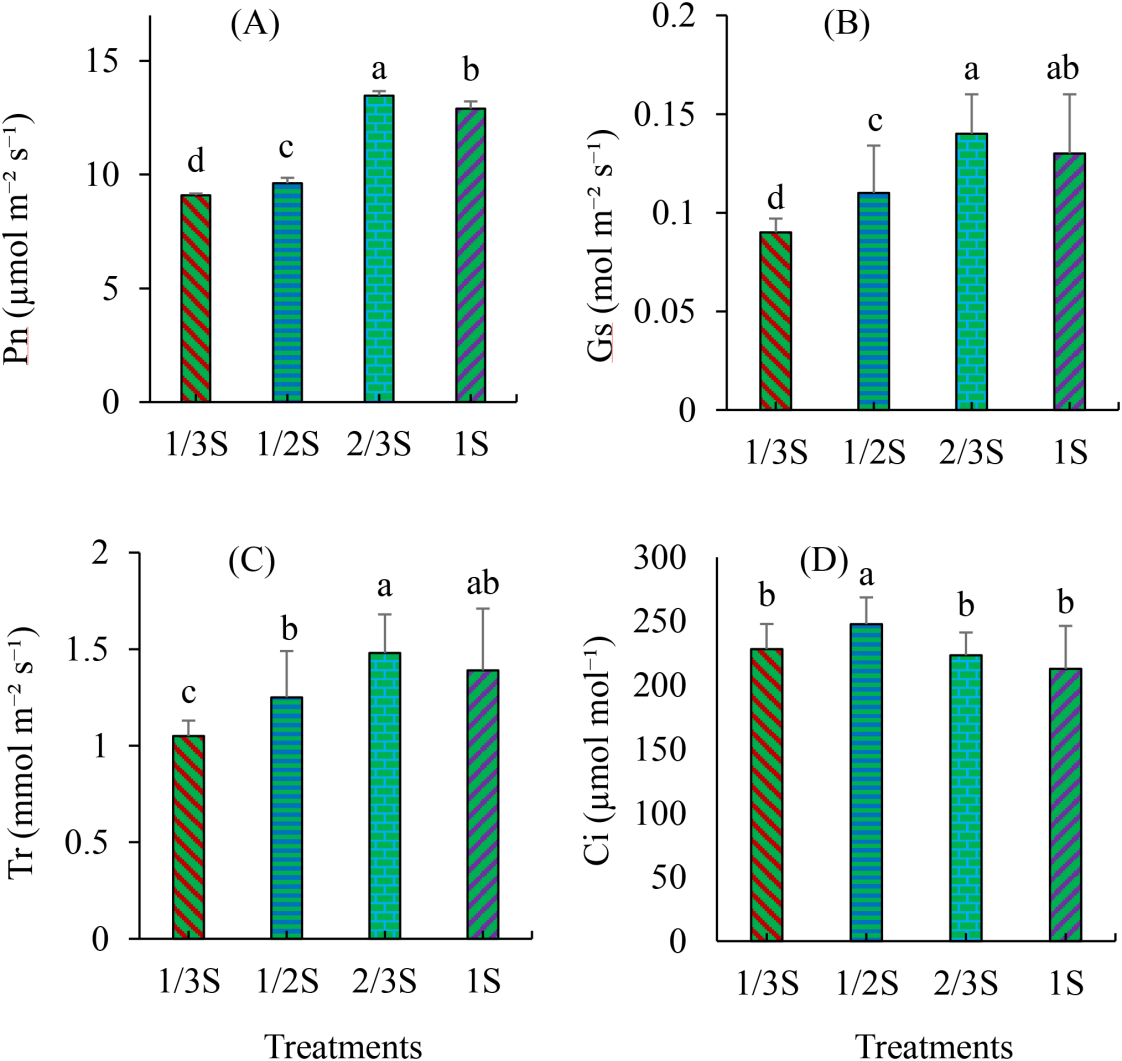
Leaf gas exchange parameters of strawberry plants subjected to different nutrient solution strengths in a recycling hydroponic system at 160 DAT. **(A)** Photosynthetic rate (Pn), **(B)** stomatal conductance (Gs), **(C)** transpiration rate (Tr), and **(D)** intercellular CO₂ concentration (Ci). Error bars represent standard deviations (n = 3). Different letters indicate statistically significant differences among treatments according to Duncan’s multiple range test (p < 0.05).

To further investigate the effects of different nutrient solution strengths on leaf gas exchange, we evaluated the net photosynthesis rate (Pn), stomatal conductance (Gs), transpiration rate (Tr), and intercellular CO2 concentration (Ci) (**Figure 2**). The Pn increased significantly with rising nutrient concentration, reaching its highest value in the 2/3S treatment, followed by the 1S and 1/2S treatments (**Figure 2A**). The lowest rate was observed in the 1/3S treatment (**Figure 2A**). Similarly, Gs was significantly higher under the 2/3S and 1S treatments compared to the 1/3S and 1/2S treatments (**Figure 2B**). The Tr was also significantly higher in the 2/3S and 1S treatments, while the 1/3S treatment had the lowest value (**Figure 2C**). The Ci showed a distinct pattern, with the highest value recorded in the 1/2S treatment, which was significantly different from those in the 1/3S, 2/3S, and 1S treatments (**Figure 2D**).

**Figure 3:**
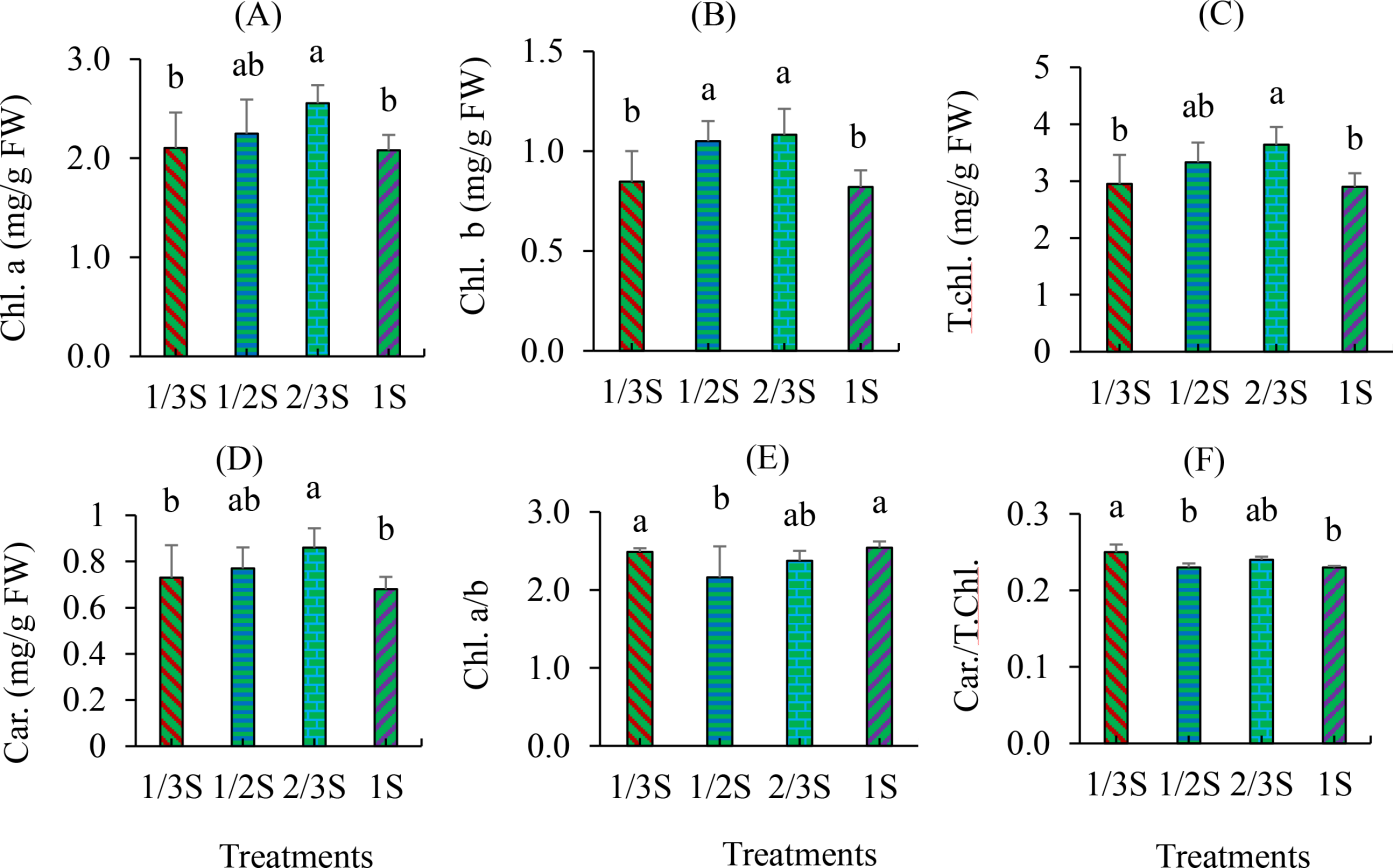
Chlorophyll and carotenoid contents in strawberry leaves subjected to different nutrient solution strengths in a recycling hydroponic system at 160 DAT. **(A)** Chlorophyll a (Chl. a), **(B)** chlorophyll b (Chl. b), **(C)** total chlorophyll (T.chl.), **(D)** carotenoids (Car.), **(E)** ratio of chlorophyll a to b (Chl. a/b), and **(F)** ratio of carotenoids to total chlorophyll (Car./T.chl.). Error bars represent standard deviations (n = 6). Different letters indicate statistically significant differences among treatments according to Duncan’s multiple range test (p < 0.05).

Moreover, nutrient solution strength significantly affected the accumulation of pigments (**Figure 3**). Chlorophyll a content was highest in the 2/3S treatment, which was statistically similar to the 1/2S treatment but significantly higher than the 1/3S and 1S treatments (**Figure 3A**). Similarly, chlorophyll b content varied significantly among treatments. The highest levels were observed in the 1/2S and 2/3S treatments, while the lowest were found in the 1/3S and 1S treatments (**Figure 3B**). Total chlorophyll content was also highest in the 2/3S treatment, which was statistically comparable to 1/2S but significantly higher than both 1/3S and 1S (**Figure 3C**). Carotenoid content was significantly higher in the 2/3S treatment compared to 1S and 1/3S, but not significantly different from the 1/2S treatment (**Figure 3D**). The chlorophyll a/b ratio was significantly higher in the 1/3S and 2/3S treatments than in the 1/2S and 1S treatments (**Figure 3E**). Additionally, the carotenoid-to-total-chlorophyll ratio was significantly higher in the 1/3S treatment compared to the 1/2S and 1S treatments, while the 2/3S treatment showed no significant difference from the others (**Figure 3F**).

### Antioxidants, leaf relative water content, electrolyte leakage, and antioxidant enzyme activities

**Figure 4:**
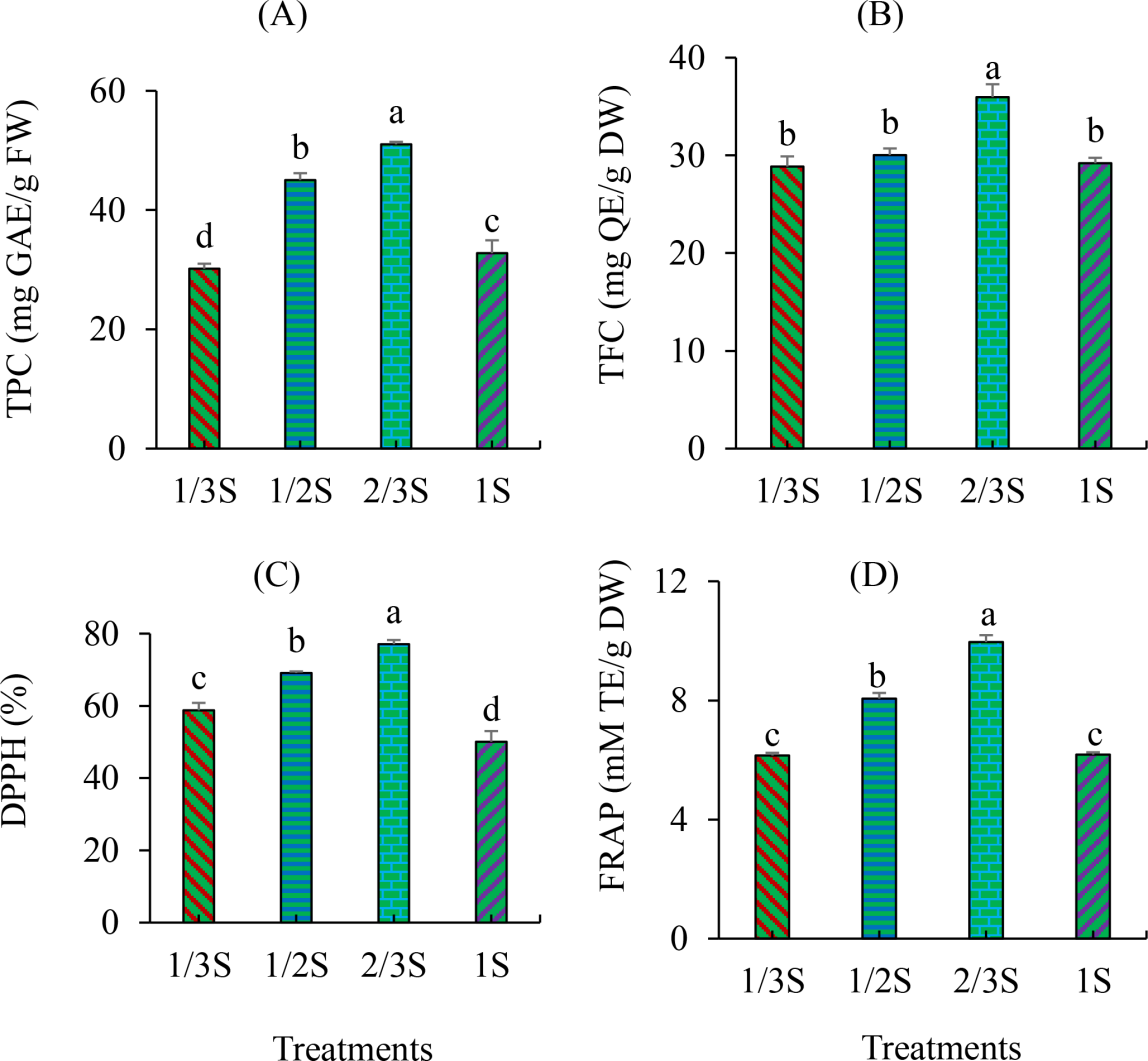
Non-enzymatic antioxidant activities in strawberry leaves subjected to different nutrient solution strengths in a recycling hydroponic system at 160 DAT. **(A)** Total phenolic content (TPC), **(B)** total flavonoid content (TFC), **(C)** DPPH radical scavenging activity (DPPH), and **(D)** ferric reducing antioxidant power (FRAP). Error bars represent standard deviations (n = 6). Different letters indicate statistically significant differences among treatments according to Duncan’s multiple range test (p < 0.05).

The effects of different nutrient solution strengths on non-enzymatic antioxidant activities in strawberry leaves are presented in **Figure 4**. Antioxidant properties varied significantly among the treatments. The highest TPC was observed in the 2/3S treatment, followed by the 1/2S (**Figure 4A**). In contrast, the lowest TPC was recorded in the 1/3S treatment, which was significantly lower than all other treatments. Similarly, the highest TFC was observed in the 2/3S treatment and was significantly higher than that in the other treatments. The lowest TFC was found in the 1S treatment, although it was not significantly different from the 1/3S and 1/2S treatments (**Figure 4B**). DPPH radical scavenging activity was also significantly affected by nutrient solution strength. The highest activity was detected in the 2/3S treatment, followed by the 1/2S and 1/3S treatments (**Figure 4C**). The lowest DPPH activity was observed in the 1S treatment. Similarly, the FRAP was highest in the 2/3S treatment, significantly greater than in all other treatments (**Figure 4D**). The lowest FRAP values were recorded in the 1/3S and 1S treatments, whereas the 1/2S treatment showed a significantly higher value than both (**Figure 4D**).

Furthermore, EL, LRWC, and antioxidant enzyme activities such as SOD, CAT, and POD were significantly affected by the treatments (**Table 7**). In this work, LRWC did not differ significantly among the treatments (**Table 7**). Regarding EL, the 1/3S and 1/2S treatments showed the highest values, while 2/3S and 1S showed significantly lower values (**Table 7**). Antioxidant enzyme activities also varied significantly among the treatments. The highest SOD activity was recorded in the 1/2S, 2/3S, and 1S treatments, whereas the lowest was observed in 1/3S (**Table 7**). In contrast, the 2/3S treatment showed the highest POD and CAT activities, while 1/3S and 1S consistently showed the lowest values for these enzymes (**Table 7**).

**Table 7:** Effects of different nutrient solution strengths on antioxidant enzyme activities of strawberry Leaves at 160 DAT.

Treatments	LRWC (%)	EL (dS·m^-1^)	SOD (U·g^-1^ FW)	POD (U·g^-1^ FW)	CAT (U·g^-1^ FW)
1/3s	82.2^a^	0.56^a^	34.6^b^	117.3^c^	41.1^d^
1/2s	80.7^a^	0.51^ab^	60.2^a^	148.0^b^	47.3^b^
2/3s	86.5^a^	0.44^b^	68.3^a^	257.3^a^	62.4^a^
1s	82.7^a^	0.43^b^	63.3^a^	112.0^c^	43.6^c^

Different letters within a column indicate statistically significant differences in means according to Duncan’s multiple range test (*p* < 0.05, n = 3). Where; LRWC, leaf relative water content; EL, electrolyte leakage; SOD, superoxide dismutase; POD, peroxidase; CAT, catalase.

### Fruit antioxidant properties and mineral content

The antioxidant activities of strawberry fruits such as TPC, TFC, FRAP and DPPH were significantly affected by the strength of the nutrient solution (**Table 8**). TPC ranged from 24.8 to 34.5 mg GAE/g DW, with the lowest value observed in the 1/3S treatment and the highest in the 2/3S treatment (**Table 8**). Similarly, TFC ranged from 10.2 to 14.1 mg QE/g DW, with the lowest value in the 1S treatment and the highest in the 2/3S treatment (**Table 8**). FRAP values ranged from 1.97 to 4.07 mM TE/g DW, with the lowest observed in the 1/3S treatment and the highest in the 2/3S treatment (**Table 8**). DPPH radical scavenging activity ranged from 69.1% to 82.5%, with the lowest value in the 1/3S treatment and the highest in the 2/3S treatment (**Table 8**). Fruit mineral content was also evaluated to observe the effects of treatments on fruit quality. The mineral content of strawberry fruits was significantly influenced by the strength of the nutrient solution (**Table 9**). Calcium content was highest in the 1/2S treatment and lowest in the 1/3S treatment (**Table 9**). Potassium concentration was higher under the 2/3S treatment, which was significantly higher than in all other treatments (**Table 9**). Magnesium content was also highest in the 2/3S treatment, while the 1/3S and 1S treatments had significantly lower values (**Table 9**). Sodium content did not vary significantly among treatments (**Table 9**). Phosphorus content was significantly higher in the 1/3S and 2/3S treatments, while the lowest value was observed under 1/2S (**Table 9**).

**Table 8:** Effects of different nutrient solution strengths on total phenolic content (TPC), total flavonoid content (TFC), ferric reducing antioxidant power (FRAP) and 2,2-diphenyl-1-picrylhydrazyl (DPPH) of strawberry fruits collected from 48 to 136 DAT.

Treatments	TPC (mg GAE/g DW	TFC (mg QE/g DW)	FRAP (mM TE/g DW)	DPPH (%)
1/3S	24.8^b^	11.5^b^	1.97^b^	69.1^b^
1/2S	28.5^ab^	11.9^ab^	2.47^b^	74.2^b^
2/3S	34.5^a^	14.1^a^	4.06^a^	82.5^a^
1S	25.2^b^	10.2^b^	2.27^b^	69.9^b^

Different letters in the column indicate statistically significant differences in means according to Duncan’s multiple range test (*p* < 0.05, n = 3).

**Table 9:** Effects of nutrient solution strengths on mineral content of strawberry fruits collected at 136 DAT.

Treatments	Calcium	Potassium	Magnesium	Sodium	Phosphorus
	mg·kg^-1^				
1/3S	1321^c^	14442^b^	1106^b^	1059^a^	2987^a^
1/2S	1735^a^	14047^b^	1239^ab^	1189^a^	2287^c^
2/3S	1589^ab^	16851^a^	1428^a^	1073^a^	2976^a^
1S	1467^bc^	13700^b^	1044^b^	1112^a^	2561^b^

Different letters in the column indicate statistically significant differences in means according to Duncan’s multiple range test (*p* < 0.05, n = 3).

### Correlation analysis between the evaluated parameters

**Figure 5:**
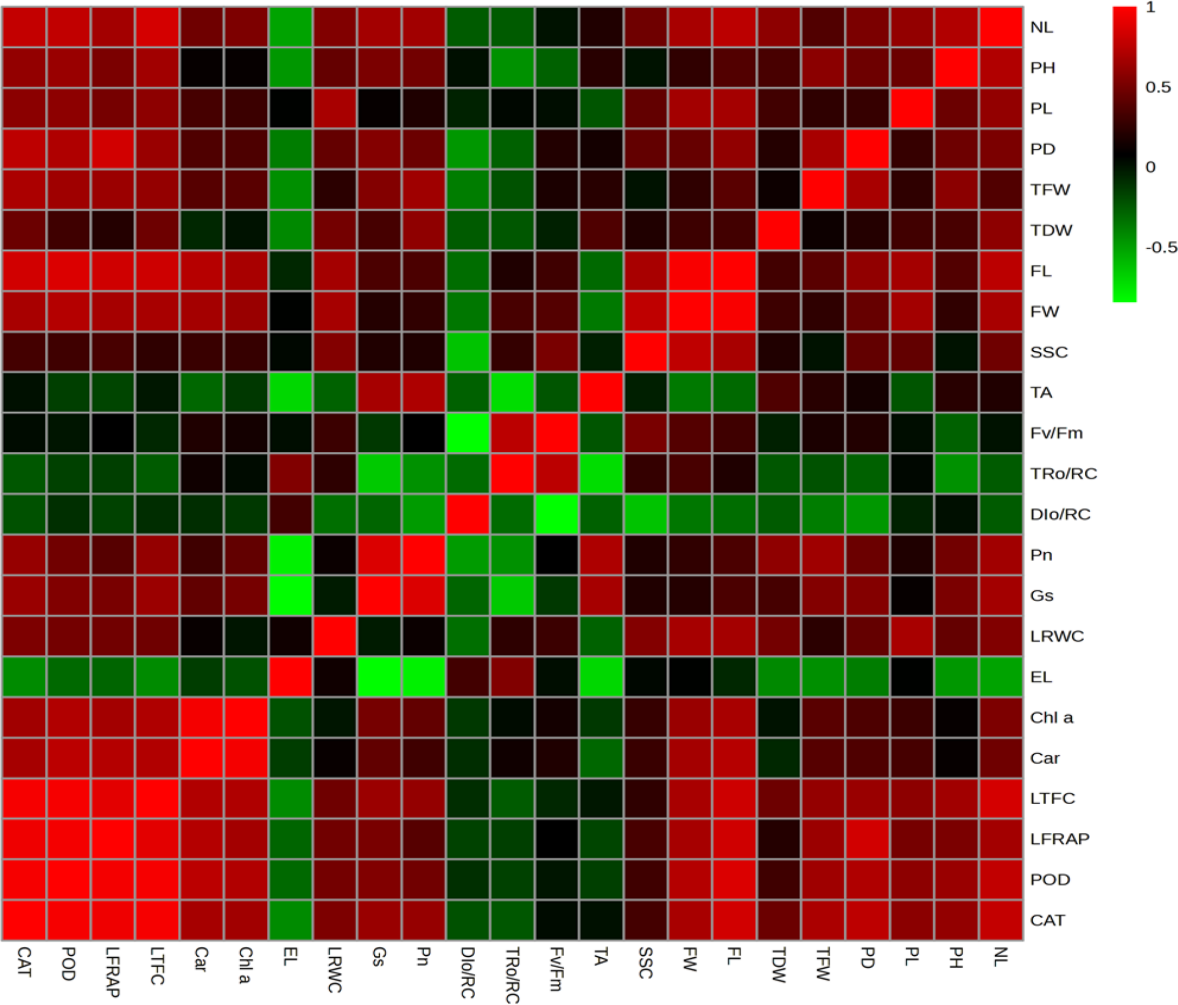
Heatmap illustrating the relationship between morphological, physiological and biochemical related parameters. Green and red colors show significant negative and positive association among the parameters, respectively. Where; NL, number of leaves; PH, plant height; PL, petiole length; PD, petiole diameter; TFW, total fresh weight; TDW, total dry weight; FL, Fruit length; FW, fruit width; SSC, soluble solid content; TA, titratable acidity; Fv/Fm, ratio of variable to maximum fluorescence; TRo/RC, trapped energy per reaction center; DIo/RC, energy dissipation per reaction center; Pn, photosynthesis rate; Gs, stomata conductance; LRWC, leaf relative water content; EL, electrolyte leakage, Chl.a, chlorophyll a; Car., carotenoids; LTFC, leaf total flavonoid content; LFRAP, leaf ferric reducing antioxidant power; POD, peroxidase, and CAT, catalase.

For the correlation analysis, we selected key parameters related to growth, physiology, biochemistry, and fruit quality to examine the relationships among them. As shown in **Figure 5**, the correlation heatmap reveals several significant positive and negative associations among the evaluated parameters. Growth parameters, such as the number of leaves (NL), plant height (PH), petiole length (PL), petiole diameter (PD), total fresh weight (TFW), and total dry weight (TDW), correlated positively with photosynthetic traits like photosynthetic rate (Pn), stomatal conductance (Gs), and leaf relative water content (LRWC). This indicates that vigorous growth is linked to higher photosynthetic capacity and improved water status. Additionally, these growth traits showed a strong negative correlation with electrolyte leakage (EL), suggesting that increased biomass is associated with reduced membrane injury across varying nutrient concentrations. Fruit morphology traits, including fruit length (FL) and fruit width (FW), positively correlated with biochemical indicators such as leaf total flavonoid content (LTFC), leaf ferric reducing antioxidant power (LFRAP), peroxidase (POD), catalase (CAT), and LRWC, implying a connection between enhanced biochemical activity and better fruit development. Conversely, titratable acidity (TA) negatively correlated with FL, FW, and EL, indicating that improved growth and lower physiological stress lead to fruits with reduced acidity. Moreover, antioxidant enzymes POD and CAT showed strong positive relationships with growth, photosynthetic efficiency, and fruit quality traits, highlighting their role in enhancing overall plant performance. In contrast, DIo/RC (energy dissipation) and TRo/RC (energy trapped per reaction center) were negatively associated with growth and photosynthetic traits, suggesting that high plant performance is linked to minimized energy loss and more efficient photochemical functioning.

### Cluster analysis

**Figure 6:**
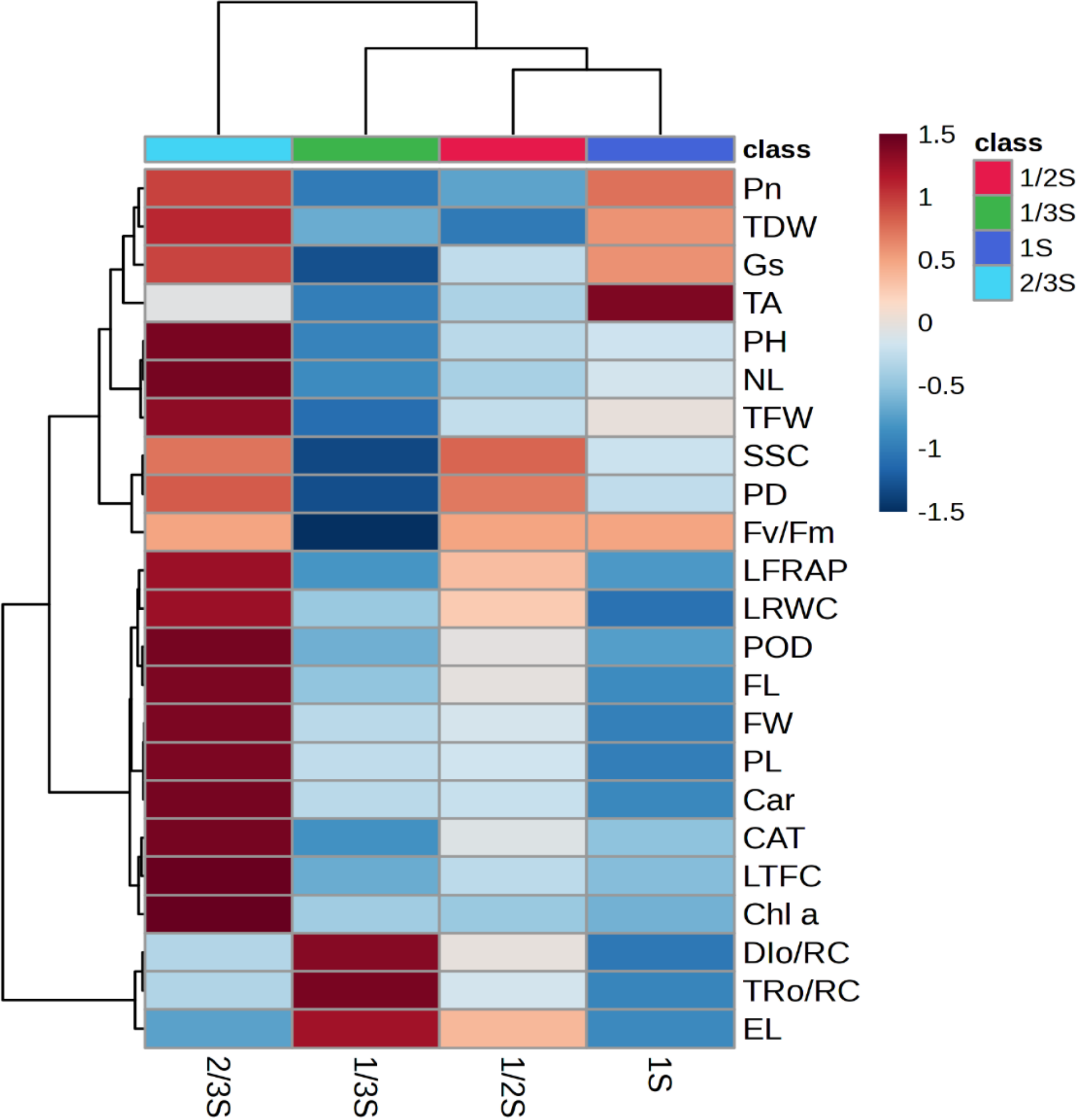
Cluster analysis illustrating the relationship between the treatments and the evaluated parameters. Different color intensities show the contribution of the parameters in each treatment as indicated on the scale bar. Where; NL, number of leaves; PH, plant height; PL, petiole length; PD, petiole diameter; TFW, total fresh weight; TDW, total dry weight; FL, Fruit length; FW, fruit width; SSC, soluble solid content; TA, titratable acidity; Fv/Fm, ratio of variable to maximum fluorescence; TRo/RC, trapped energy per reaction center; DIo/RC, energy dissipation per reaction center; Pn, photosynthesis rate; Gs, stomata conductance; LRWC, leaf relative water content; EL, electrolyte leakage, Chl.a, chlorophyll a; Car., carotenoids; LTFC, leaf total flavonoid content; LFRAP, leaf ferric reducing antioxidant power; POD, peroxidase, and CAT, catalase.

To illustrate the differences among treatments and identify the parameters that contributed most to the observed variation, a cluster analysis was conducted using selected key traits. The resulting hierarchical clustering clearly separated the four nutrient-solution strengths, as shown in **Figure 6**. The variables were organized into three main clusters. The first cluster included parameters related to photosynthetic activity and vegetative growth: net photosynthetic rate (Pn), stomatal conductance (Gs), total dry weight (TDW), total fresh weight (TFW), plant height (PH), number of leaves (NL), petiole diameter (PD), titratable acidity (TA), soluble solids content (SSC), and maximum quantum efficiency of PSII (Fv/Fm). These traits indicated plant vigor and fruit quality. The second cluster consisted of biochemical and pigment-related traits, including leaf total flavonoid content (LTFC), leaf ferric reducing antioxidant power (LFRAP), peroxidase (POD), catalase (CAT), carotenoids (Car), chlorophyll *a* (Chl *a*), and leaf relative water content (LRWC). This group also included fruit morphological parameters such as fruit length (FL), fruit width (FW), and petiole length (PL), reflecting metabolic activity, stress defense, pigment accumulation, and water status. The third cluster included stress-associated parameters: trapped energy per reaction center (TRo/RC), dissipated energy per reaction center (DIo/RC), and electrolyte leakage (EL), linked to photoinhibition and membrane damage under stress.

The clustering pattern differentiated treatments based on growth, physiological, biochemical, and fruit traits. Plants grown under the lowest nutrient strength (1/3S) aligned with the stress-related cluster, exhibiting elevated TRo/RC, DIo/RC, and EL, indicating higher physiological stress. In contrast, plants with the full-strength nutrient solution (1S) demonstrated higher titratable acidity (TA) along with moderate values of Pn, Gs, and TDW. The 1/2S treatment was associated with moderate levels of SSC, Fv/Fm, and PD, suggesting intermediate physiological performance. Plants grown under 2/3S were linked to enhanced growth traits (PH, NL, PL, and TFW) and exhibited higher Pn and Gs. This nutrient strength also clustered with most biochemical and pigment-related traits (LTFC, LFRAP, POD, CAT, Car, and Chl a), indicating increased antioxidant capacity and pigment accumulation. Additionally, fruit size traits (FL and FW) were associated with 2/3S, demonstrating that this treatment supported superior fruit development.

### Principal component analysis

**Figure 7:**
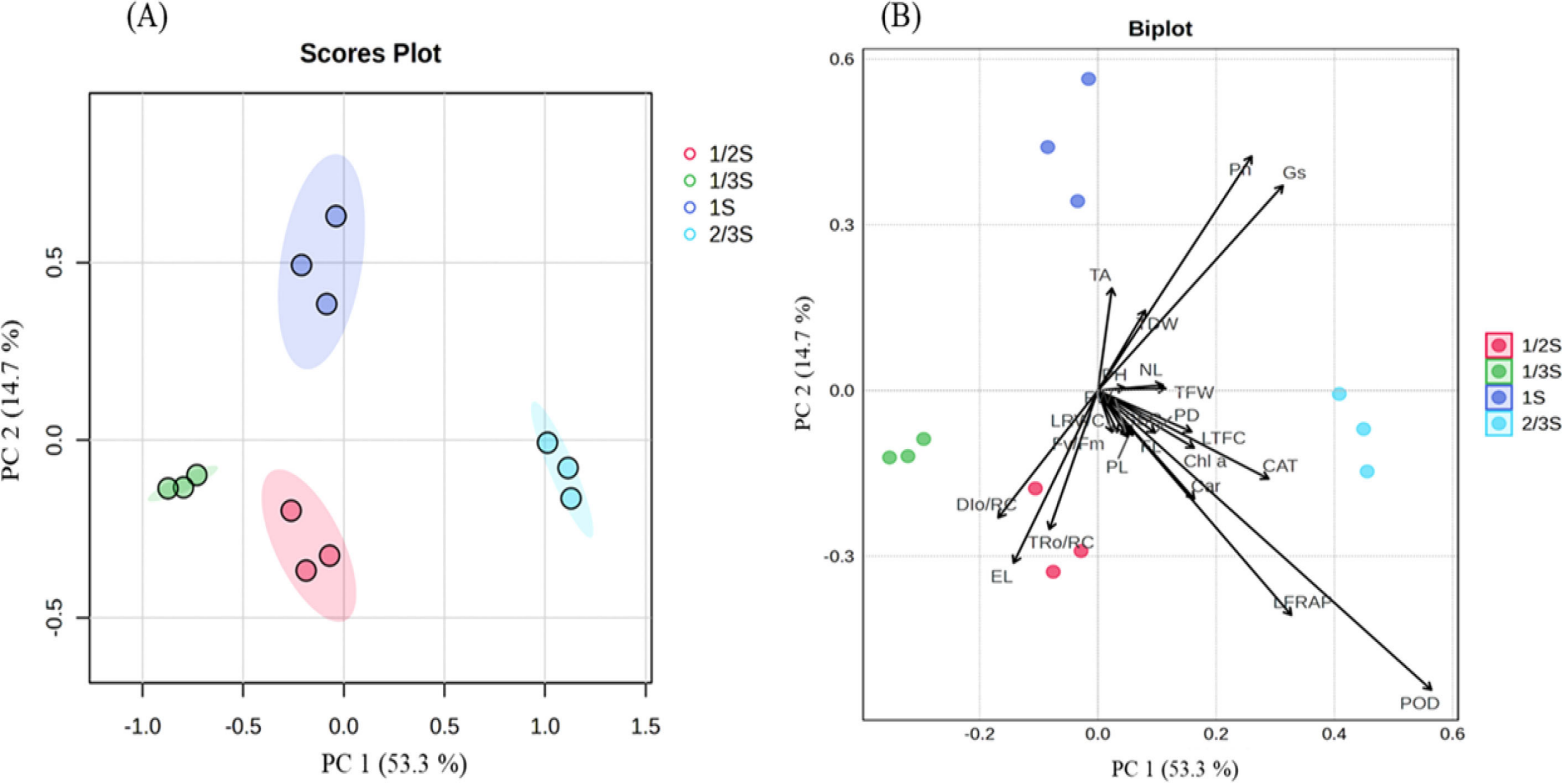
**(A)** Scores plot showing a distinct separation among the treatments and **(B)** Biplot illustrating the relationships between treatments and various physiological, biochemical, and morphological traits of strawberry plants. Where; NL, number of leaves; PH, plant height; PL, petiole length; PD, petiole diameter; TFW, total fresh weight; TDW, total dry weight; FL, Fruit length; FW, fruit width; SSC, soluble solid content; TA, titratable acidity; Fv/Fm, ratio of variable to maximum fluorescence; TRo/RC, trapped energy per reaction center; DIo/RC, energy dissipation per reaction center; Pn, photosynthesis rate; Gs, stomata conductance; LRWC, leaf relative water content; EL, electrolyte leakage, Chl.a, chlorophyll a; Car., carotenoids; LTFC, leaf total flavonoid content; LFRAP, leaf ferric reducing antioxidant power; POD; peroxidase, and CAT, catalase.

To more clearly illustrate the relationships between the treatments and the measured parameters, we performed a principal component analysis (PCA). The scores plot (**Figure 7A**) showed clear distinctions among the four nutrient treatments, indicating significant differences in physiological, biochemical, and morphological performance. The biplot (**Figure 7B**) highlighted factors contributing to this separation, demonstrating how specific traits influenced PCA distribution. Growth-related traits such as number of leaves, plant height, petiole length, total fresh weight, and total dry weight were closely aligned with the 1S and 2/3S clusters, suggesting that higher nutrient strengths enhanced vegetative growth and biomass accumulation. Photosynthetic parameters, including photosynthetic rate and stomatal conductance, were associated with these treatments, indicating improved photosynthetic efficiency. Chlorophyll-related traits (chlorophyll *a* and carotenoids) and antioxidant indicators, such as leaf total flavonoid content, leaf ferric reducing antioxidant power, catalase, and peroxidase, were linked to the 2/3S treatment, suggesting better management of oxidative stress. In contrast, traits indicative of physiological stress, such as electrolyte leakage and energy dissipation per reaction center, clustered with the 1/3S treatment, highlighting that nutrient deficiency led to increased cellular damage. Fruit quality attributes, including soluble solid content, fruit width, and titratable acidity, were associated with moderate to high nutrient treatments, reinforcing the benefits of sufficient nutrient availability on berry development. Plants receiving 2/3S exhibited superior physiological stability, photosynthetic activity, antioxidant capacity, and growth, while those at 1/3S displayed signs of stress. The distinct clustering patterns and trait loadings underscore the importance of providing an optimal nutrient solution to maximize growth, health, and fruit quality of the ‘Kuemsil’ strawberry cultivar in recycling hydroponic systems.

## Discussion

Plants adapt to nutrient availability through morphological, physiological, and biochemical adjustments that ultimately influence growth and yield. Nutrient solution concentration is a key factor influencing plant growth and fruit quality, with numerous studies highlighting its significant effects on performance. For example, Tohidloo et al. (2018) reported that a potassium concentration of 350 mg·L^−^¹ maximized leaf area, shoot biomass, yield, and vitamin C content, whereas 600 mg·L^−^¹ often reduced quality traits in different strawberry cultivars. Similarly, Yousefi et al. (2023) found that applying a K/N ratio of 200/180 mg·L^−^¹ during the vegetative stage, followed by 300/120 mg·L^−^¹ in the reproductive stage, optimized fruit yield, soluble sugar, and starch content. In contrast, maintaining consistently high K and N levels (300/180 mg·L^−^¹) favored vegetative growth and photosystem II efficiency.

In this study, we evaluated morphological traits including the number of leaves, petiole width, leaf length, crown diameter, and total fresh weight. Significantly higher values for these traits were observed in plants treated with 2/3S and 1/2S nutrient solutions, while the 1/3S treatment consistently showed the lowest growth performance (**Table 4**). The highest vegetative growth under 2/3S is associated with enhanced photosynthetic activity, improved gas exchange, and increased chlorophyll content (**Figure 6**). Increased vegetative growth enlarges leaf surface area and source strength, promoting the assimilation and translocation of photoassimilates to support fruit development. Reduced vegetative growth, however, declines physiological functions, negatively impacting the biochemical composition and fruit quality of strawberries. These findings support previous studies by Lee J. Y, et al. (2017) and Nazarideljou et al. (2019), which indicated that appropriate nutrient concentrations stimulate cell division, expansion, and organ development, improving growth performance and fruit characteristics. In addition, the highest phosphorus concentration was observed in the leaves of plants grown under the 2/3S treatment (data not shown). Phosphorus is essential for energy transfer, cell division, and nucleic acid synthesis (Khan et al., 2023), and its elevated accumulation may have contributed to the superior vegetative growth recorded in this treatment. Similarly, the highest calcium content was also detected in the leaves of plants grown under the 2/3S treatment (data not shown). Calcium is vital for maintaining cell wall structure, stabilizing cell membranes, and regulating signaling pathways involved in growth and stress responses (Thor, 2019). Its increased accumulation possibly enhanced structural integrity and maintained cellular function, thereby supporting the improved growth performance observed under the 2/3S treatment.

OJIP-fluorescence parameters are valuable indicators of PSII functional status and overall photosynthetic performance under varying environmental and nutritional conditions (Bussotti et al., 2020). Different strengths of nutrient solutions significantly influenced the OJIP parameters (**Table 6**). The 1/3S treatment significantly reduced PSII efficiency as indicated by lower Fv/Fm, Fv/Fo, and PiAbs values. In this investigation, the decline in photochemical efficiency under the 1/3S treatment is associated with reduced vegetative growth and inhibited photosynthetic activity (**Figure 6**). In contrast, plants treated with 1/2S, 2/3S, and 1S nutrient solution showed significantly higher Fv/Fm, Fv/Fo, and PiAbs values, indicating optimal energy use efficiency. Interestingly, ABS/RC, TRo/RC, and ET_0_/RC were significantly higher in the 1/3S treatment than in the others (**Figure 6**). This increase may be attributed to over-excitation of remaining active reaction centers under 1/3S treatment, which may be due to degradation of some PSII units (Kumar et al., 2020). However, this elevated energy flux did not translate into increased photochemical efficiency, as shown by lower Fv/Fm and PiAbs values. The increase in DIo/RC under 1/3S treatment indicates higher energy loss as heat, suggesting activation of non-photochemical quenching (NPQ) mechanisms to prevent photodamage under stress (Stefanov et al., 2022). Moreover, leaf gas exchange parameters, including net photosynthetic rate, stomatal conductance, and transpiration rate, were highest in the 2/3S treatment, aligning with chlorophyll fluorescence and growth performance. The observed reduction in intercellular CO_2_ concentration under 2/3S indicates efficient CO_2_ fixation and higher photosynthetic capacity. In the 1/3S treatment, both the photosynthetic rate and intercellular CO_2_ concentration were low. This may be due to low stomatal conductance and metabolic limitations resulting from suboptimal nutrient availability. Similarly, a reduction in intercellular CO_2_ concentration in strawberry leaves was observed under different nutrient solution concentrations when photosynthesis rates were high (Li et al., 2013). Enhanced water status and membrane stability, indicated by higher chlorophyll content (**Figure 3**) and lower EL (**Table 7**), further contributed to the higher photosynthetic performance of the 2/3S treated plants. These physiological responses indicate improved stomatal regulation and metabolic activity, critical for sugar synthesis and allocation to developing fruits (Rahman et al., 2022). Furthermore, the highest concentration of magnesium in the leaves was observed in plants treated with the 2/3S nutrient solution (data not shown). Magnesium plays a crucial role in promoting healthy leaf development, which in turn enhances leaf gas exchange and other essential physiological processes (Ahmed et al., 2023). Therefore, the improved OJIP and leaf gas exchange parameters observed under the 2/3S treatment may also be attributed to the elevated levels of this mineral.

Chlorophyll and carotenoids are essential for photosynthesis and serve as indicators of plant health (Izadpanah et al., 2024). The present findings showed that different nutrient solution strengths significantly affected the accumulation of chlorophyll a, chlorophyll b, total chlorophyll, and carotenoids in strawberry leaves (**Figure 3**), highlighting the importance of nutrient availability in photosynthetic pigment biosynthesis. Chlorophyll a, b, and total chlorophyll levels increased significantly in the 2/3S and 1/2S treatments, supporting better chloroplast development. In contrast, the 1/3S treatment showed lower pigment content and impaired PSII function, which reduced photosynthetic rates. Furthermore, carotenoid levels and chlorophyll a/b ratios indicate photoprotection and energy distribution. The higher carotenoid content in 2/3S treated plants enhances photoprotection and reduces oxidative stress (Zahedi et al., 2023). The high chlorophyll a/b ratio in the 1/3S and 1S treatment may have resulted from a decrease in chlorophyll b. Moreover, plants under the 2/3S treatment had a higher carotenoid-to-total chlorophyll ratio, indicating improved photosynthetic efficiency and enhanced photoprotection (Yoo et al., 2003). On the other hand, the increased ratio in the 1/3S treatment indicates a significant reduction in chlorophyll content than carotenoid levels. Chlorophyll and carotenoid contents are also key indicators of plant resilience and light-use efficiency (Muñoz and Munné-Bosch, 2018). The optimal pigment profile in 2/3S treatment suggests a favorable nutrient balance that supports pigment biosynthesis without causing nutrient stress.

Antioxidants such as phenols and flavonoids are critical secondary metabolites that enhance plant resilience against oxidative stress (Rao and Zheng, 2025). This study found that nutrient solution strength significantly influenced the antioxidant capacity of strawberry leaves (**Figure 4**). Among all treatments, the 2/3S plants showed the highest antioxidant activity. In particular, TPC and TFC levels were significantly elevated compared to the other treatments. This suggests that the 2/3S treatment enhances the biosynthesis of secondary metabolites, which are essential for scavenging ROS and mitigating oxidative stress (Chaves et al., 2017). Both 1/3S and 1S treatments showed lower antioxidant properties. This may be due to reduced activation of the polyphenolic biosynthetic pathway or increased oxidative damage. These findings are consistent with Narvekar and Tharayil (2021), who reported enhanced phenolic metabolism under optimal nutrient conditions. Moreover, DPPH and FRAP activities were higher in the 2/3S plants. This further supports the conclusion that the 2/3S nutrient strength creates a favorable physiological environment for stronger antioxidant defense. Overall, these results indicate that 2/3S not only promotes the biosynthesis of antioxidant compounds but also enhances their functional activity in scavenging free radicals and reducing oxidative damage.

Nutrient solution strength also influenced LRWC, EL, and antioxidant enzyme activities, including SOD, POD, and CAT (**Table 7**). Although LRWC did not differ significantly among treatments, the numerically higher LRWC in the 2/3S treatment suggests improved osmotic adjustment and water retention. EL is a common indicator of membrane damage associated with oxidative stress (Demidchik et al., 2014). Both 1/3S and 1/2S treatments showed significantly higher EL values compared to other treatments, indicating that inadequate nutrient supply may negatively affect membrane integrity in the ‘Kuemsil’ strawberry cultivar. This could result from an imbalance in nutrient uptake, leading to cellular stress and ROS generation. On the other hand, the 2/3S and 1S treatments showed the lowest EL value, indicating enhanced membrane stability and reduced oxidative damage. Antioxidant enzymes such as SOD, POD, and CAT are crucial for mitigating oxidative damage by detoxifying ROS (Rao et al., 2025). SOD catalyzes the dismutation of O_2_·− into H_2_O_2_ and O_2_, serving as the first line of defense against oxidative stress (Zebro and Heo, 2024b). POD and CAT further decompose H_2_O_2_ into water and oxygen, preventing cytotoxic accumulation (Singh, 2022). In this finding, SOD activity was significantly enhanced in the 1/2S, 2/3S, and 1S treatments, with the highest value in 2/3S, indicating that 2/3S treatment supports the upregulation of antioxidant responses, thereby reducing oxidative stress. The 1/3S treatment had the lowest SOD activity, reflecting a weakened defense response. Similarly, POD and CAT activities were highest in the 2/3S treatment and lowest in the 1/3S and 1S treatments. This suggests that while 1S may not cause membrane damage (**Table 7**), it may not sufficiently stimulate the antioxidant system, potentially due to nutrient-induced metabolic shifts that suppress enzyme activities (Ihtisham et al., 2023). The highest performance of plants treated with 2/3S across all antioxidant enzyme activities suggests an optimal balance between nutrient availability and metabolic regulation. Moreover, in this work, the antioxidant enzyme profiles are consistent with patterns observed in chlorophyll fluorescence (**Table 6**), chlorophyll content and carotenoid content (**Figure 3**) and antioxidants (**Figure 4**), underscoring the importance of antioxidant systems in maintaining physiological stability under this specific nutrient solution strength. A study by Neocleous et al. (2012) also reported that increased antioxidant enzyme activity is a strong indicator of enhanced stress tolerance.

Fruit quality, a critical determinant of strawberry marketability, was also influenced by nutrient solution strength (**Table 5**). The 2/3S treatment consistently produced superior fruit quality, including higher average fruit weight, larger size (length and width), higher firmness, and increased SSC. These improvements were associated with enhanced vegetative growth and more efficient antioxidant enzyme activity (**Figure 6**). In particular, firmness and SSC reflect structural integrity and sugar accumulation, both of which are highly sensitive to the plant’s physiological condition. Although the 1S treatment increased TA, it did not enhance other fruit quality attributes, suggesting potential negative effects of this nutrient solution strength. These findings align with Rosado Souza et al. (2023), who reported that excess nutrients can disrupt the source-sink balance and reduce fruit quality. Furthermore, the concentration of antioxidants in strawberry fruits is crucial for assessing fruit quality and nutritional value. This study found that the antioxidant activities of strawberry fruits were significantly influenced by the strength of the nutrient solution (**Table 8**). The 2/3S treatment consistently demonstrated the highest antioxidant capacity, emphasizing the importance of optimizing nutrient supply for enhancing strawberry quality. The high antioxidant content in 2/3S fruits improves nutritional quality, playing a significant role in reducing chronic diseases (Çeliktopuz et al., 2025). However, high accumulation of phenolic compounds, such as flavonoids, is often associated with bitterness or astringency, which reduces the taste quality of the fruits and may compromise consumer acceptance. Interestingly, in this study, the treatment with the highest antioxidant properties also showed the highest SSC. Since sweetness generally dominates taste perception, the increased soluble solids likely compensated for any bitterness, thereby maintaining or even improving sensory quality. In contrast, the 1/3S and 1S treatments showed significantly lower antioxidant activity, indicating that both the lowest and highest nutrient concentrations can negatively impact fruit quality. These results align with Nazarideljou et al. (2019), who reported that nutrient solution strength significantly affects fruit quality and nutraceutical properties.

Moreover, the observed variations in fruit mineral content across the different nutrient solution strengths indicate that mineral uptake in strawberries is highly sensitive to nutrient concentration adjustments in the hydroponic system. The lower levels of potassium and magnesium in the 1/3S and 1S treatments suggest that both 1/3S and 1S solutions may hinder optimal uptake of these minerals, which may be due to imbalances in nutrient availability or competition among cations at the root interface. The lowest calcium concentration in the 1/3S treatment further supports the idea that insufficient nutrient supply limits the transport of essential minerals to the fruit. In contrast, the 1/2S treatment resulted in elevated accumulation of calcium and magnesium, while concurrently reducing phosphorus content. This inverse relationship is indicative of nutrient antagonism, wherein increased calcium availability may inhibit phosphorus uptake by competing for root transport mechanisms, as described in Elmendorf and Pierce (1940). Furthermore, Lee J.Y. et al. (2017) reported an antagonistic relationship between these two minerals in *Solanum lycopersicum* grown under hydroponic conditions. Interestingly, the 2/3S treatment produced a more balanced mineral profile, with improved potassium, magnesium, and phosphorus concentrations, suggesting that moderate nutrient solution strength may optimize mineral uptake efficiency. Such improvements in fruit mineral composition are not only beneficial for plant physiological performance but also enhance the nutritional quality of strawberries, contributing to human dietary mineral intake (Ali, 2023).

To sum up, the evaluated ‘Keumsil’ variety demonstrated optimal growth and physiological vigor in a nutrient solution with an EC of 1.9 dS·m^-1^ (2/3S), indicating its tolerance and adaptability to higher EC conditions. In contrast, previous studies indicated that most varieties grown in Korea flourish at relatively low EC levels. For instance, the ‘Maehyang’ variety performs best at an EC of 0.8 to 1.2 dS·m^-1^ (Jun et al., 2013). On the other hand, the ‘Seolhyang’ variety shows better adaptability to moderately higher EC levels, with optimal growth and fruit quality achieved between 0.8 and 1.5 dS·m^-1^ (Lee et al., 2019). The broader adaptability ranges of ‘Keumsil’ underscore its potential for advanced hydroponic cultivation.

## Conclusion

Strawberry production in recycling hydroponic systems requires precise nutrient management to optimize plant growth and fruit quality while minimizing stress and resource waste. This study found that varying nutrient solution strengths significantly influenced the growth, photosynthetic efficiency, antioxidant capacity, and fruit quality of the ‘Kuemsil’ strawberry cultivar. The 2/3S nutrient solution (EC 1.9 dS·m^−^¹) consistently produced the best results, including greater vegetative vigor, higher photosynthetic activity, increased antioxidant levels, and improved fruit quality traits such as sweetness, firmness, and nutritional value. These findings suggest that the 2/3S solution is ideal for growers aiming to balance resource efficiency with high productivity in recycling hydroponic systems. Its reduced nutrient concentration supports strong plant performance, lowers fertilizer costs, and decreases the risk of nutrient accumulation, which is important for long-term system stability. Additionally, adopting a slightly lower nutrient strength can enhance the sustainability of hydroponic operations by reducing input use and minimizing environmental impacts. However, limitations such as nutrient accumulation in closed-loop systems, environmental fluctuations, and cultivar-specific responses may affect long-term performance. Ongoing monitoring of solution EC, ion balance, and plant physiological indicators is essential. Future research should evaluate long-term sustainability and economic feasibility under commercial-scale conditions to refine nutrient management strategies for high-quality ‘Kuemsil’ strawberry production.

## Data Availability

The original contributions presented in the study are included in the article/**Supplementary Material**, further inquiries can be directed to the corresponding author/s.

## Supplementary material

Supplementary Table 1 Pearson correlation coefficients showing the relationships among the evaluated parameters. Where, * indicates p,0.05, ** indicates P<0 01 and *** indicates p<0.001.
